# Assessment of the Levels of Airborne Bacteria, Gram-Negative Bacteria, and Fungi in Hospital Lobbies

**DOI:** 10.3390/ijerph10020541

**Published:** 2013-01-31

**Authors:** Dong-Uk Park, Jeong-Kwan Yeom, Won Jae Lee, Kyeong-Min Lee

**Affiliations:** 1 Department of Environmental Health, Korea National Open University, Seoul, 110-791, Korea; 2 Hanbul Energy Manufacturing, Sampyeong-Dong, Bundang-Gu, Gyeonggido, 463-413, Korea; 3 Department of Global Healthcare Management, Gachon University, Gyeonggido, 461-701, Korea; 4 Institute for Occupational Health & Graduate School of Public Health, Yonsei University, Seoul, 120-752, Korea

**Keywords:** hospital lobby, bacteria, fungi, Gram-negative bacteria (GNB)

## Abstract

*Aims:* We assessed the levels of airborne bacteria, Gram-negative bacteria (GNB), and fungi in six hospital lobbies, and investigated the environmental and hospital characteristics that affected the airborne microorganism levels. *Methods:* An Andersen single-stage sampler equipped with appropriate nutrition plate agar was used to collect the samples. The three types of microorganisms were repeatedly collected at a fixed location in each hospital (assumed to be representative of the entire hospital lobby) from 08:00 through 24:00, with a sampling time of less than 5 min. Temperature and relative humidity were simultaneously monitored. *Results:* Multiple regression analysis was used to identify the major factors affecting microorganism levels. The average levels of bacteria (7.2 × 10^2^ CFU/m^3^), GNB (1.7 × 10 CFU/m^3^), and fungi (7.7 × 10 CFU/m^3^) indicated that all hospital lobbies were generally contaminated. Season was the only factor that significantly affected the levels of all microorganisms (*p* < 0.0001), where contamination was the highest during the summer, significantly higher than during the winter. Other significant factors varied by microorganism, as follows: airborne bacteria (number of people in the lobby, sampling time), GNB (scale of hospital), and fungi (humidity and air temperature). *Conclusions:* Hospital lobby air was generally contaminated with microorganisms, including bacteria, GNB, and fungi. Environmental factors that may significantly influence the airborne concentrations of these agents should be managed to minimize airborne levels.

## 1. Introduction

Exposure to biological agents (including microorganisms) is associated with a wide range of major public health issues, such as infectious diseases, acute toxic effects and allergies [1]. Although airborne microorganisms encountered in hospital lobbies are apparently harmless to healthy people, they can cause adverse health effects in immune-compromised people. Many of those who pass through hospital lobbies belong to the vulnerable group of weak, elderly, and infirm people, and thus may be very sensitive to biological hazards. In particular, hospitalized patients could be at significantly increased risk of bio-aerosol exposure [2].

Airborne microorganisms originate not only from people (including patients), but are also spawned by various indoor hospital characteristics and outdoor environmental sources. There are a number of such factors that may be related to the generation of bio-aerosols in the lobby of a hospital. Airborne microorganisms and other sources of contamination in hospitals must be reduced to a minimum, because many of the people passing through hospital lobbies could be very sensitive to these hazardous agents. Thus, to maintain the lowest possible airborne microorganism levels in hospital lobbies, it is crucial to identify the factors influencing these levels. Hospital buildings may be regarded as dynamic environments affected by season, weather conditions, indoor ventilation system design and operation, intrusion of moisture, the outdoor microbial load and the number of occupants and visitors, and human activities. These factors can be related to a condition for microbial growth. Nutrient availability, indoor temperature, and shelter generally cannot be managed to limit indoor microbial survival and growth. Several studies have been conducted to determine the factors influencing microbial growth in hospital environments. Scaltriti *et al.* reported that risk factors for microbial contamination of air in operating theatres were the frequency of door-opening, taken as an index of staff and visitor movement, was a positive predictor of raised bacterial counts [3]. Obbard *et al.* identified the occupant density as the key factor influencing the level of airborne bacteria. Humidity was also found to be important factor depending on the particular location within the hospital [4].

To date, airborne microorganism levels have been reported in hospital clean rooms, such as operating rooms, hospital rooms, intensive care units, surgical units, hematological wards, and maternity wards [5,6,7]. To the best of our knowledge, no previous studies have investigated how hospital characteristics are associated with airborne levels of microorganisms in lobbies. Accordingly, we examined the levels of airborne bacteria, gram negative bacteria (GNB), and fungi with respect to environmental and other characteristics in the lobbies of six hospitals. 

## 2. Methods

### 2.1. Monitoring Strategy

Microorganism levels, relative humidity, and temperature were measured in six Korean hospitals with more than 500 beds, between July (summer) and January (winter). General characteristics for general hospital lobby were shown in Table 1. We are unaware of any hospital that has installed carpet on the entire hospital floor (including the lobby) in Korea. No hospitals have a wooden construction or use other materials that would provide a good environment for microbial growth. A different hospital was examined every month. Air samples were repeatedly taken from the start of lobby service (08:00 AM) through the end of lobby service (18:00 PM), continuing until midnight (24:00). The air was monitored after lobby service hours for comparison with samples taken during lobby service hours. Sampling instruments were placed at a height of around 1.2–1.5 m, in a fixed location assumed to be representative of the entire lobby. 

**Table 1:** General characteristics for the hospitals investigated

Hospital	No of bed	Building age, year	Area of lobby (m^2^)
A	710	23	2,764
B	1,000	10	2,717
C	1,200	3	2,413
D	530	26	1,674
E	867	19	2,712
F	800	13	1,710

Our microbial sampling strategy was designed to best represent the level of microorganisms generated in hospital lobby environment. An Anderson single-stage cascade sampler was used for all collections, with a flow rate of 28.3 L/min and a sampling time of less than 5 min to avoid the collection of unaccountable microorganisms. The airborne microorganisms were targeted one after another, using a 20 mL nutrient plate (tryptic soy agar, TSA) for bacteria, MacConkey agar (MAC) for GNB, and sabouraud dextroseagar with chloramphenicol (SDAC) for fungi) coupled inside the stage sampler. When changing the collection plates, the stage hole was sterilized with a 70% ethanol solution to prevent cross-contamination. To evaluate the representative level of airborne microorganisms during the morning (08:00–13:00), afternoon (13:00–18:00) and after lobby service (18:00–24:00), three or four samples by type of microorganism were those taken in each respective period. The sampling interval between types of microorganisms was the duration required to both change the collection plates and take samples for the other two types of microorganisms. After sample collection, the agar plates were transported to the laboratory and incubated at 37 ± 1 °C for 2 days for bacteria and GNB and at 25 ± 1 °C for 4 days for fungi [8]. Specific statistical corrections were made using correction factors obtained for each growing medium from Conversion Tables provided by single stage operating manual [9]. For all the media the minimum detectable number of colony forming units (CFU) was 30 per sample. Data below minimum detectable number was treated as missing. The microbial counts were expressed in terms of colony-forming units (CFU) per unit volume of air (m^3^). Total culturable bacterial and fungal counts were compared to ambient environmental levels (background) to determine the degree of incremental levels generated during household waste handling. Our microbial sampling strategy mentioned above was determined to best represent the level of microorganisms generated in hospital lobby. Direct-reading instruments for monitoring the temperature and humidity (IAQ, Model; 8762, TSI, Shoreview, MN, USA) were placed next to the cascade sampler to simultaneously monitor air temperature and humidity during each sampling interval. 

### 2.2. Data Analysis

The data-logging interval was set at 30 s, averaged with a time-weighted average. Direct-reading temperature, humidity, and CO_2 _results were matched with airborne microorganism levels measured during the sampling period of microorganisms. We selected a number of environmental variables and hospital characteristics that may influence airborne microorganism levels in hospital lobbies. The quantitative variables were categorized based on subjective classification, as follows: the scale of a hospital expressed by the number of beds (<1,000 *vs.* ≥1,000), lobby age (<10 years, 10–19 years, and ≥20 years), the input location of outdoor air in the HVAC (Heating, Ventilating and air conditioning) system (underground *vs.* ≥1st floor), the number of filters in the HVAC system (pre and medium *vs.* only medium), and lobby volume (<10,000 m^3^ *vs.* ≥10,000 m^3^). Both lobby service time and cleaning time were dichotomized into “on (08:00–18:00)” and “off (18:00–24:00)”. Correlations between the selected independent variables were evaluated to identify potential problems prior to offering to the multiple regression model. Correlations involving at least one continuous variable were tested using Spearman’s rho, and between two dichotomous variables using Kendall’s tau. The absolute values of all correlation coefficients were less than 0.5, so all of the above variables were considered appropriate for input to a single model.

Simple linear regression analysis was used to examine the relationships among quantitative variables such as air temperature, humidity, CO_2_ level, and the number of people in the lobby during sampling, and microbial concentrations. Analysis of variance (ANOVA) was employed to compare mean microorganism levels between categories of qualitative variables. Only variables with p-values less than 0.25 were ultimately included in the multiple regression analysis [10]. Stepwise analysis was used to identify the final factors that significantly affected the airborne concentrations of microorganisms. Descriptive statistics were obtained and data analysis was conducted using the STATA (Version 11.0) software (Stata Corp. LP, College Station, TX, USA).

The concentrations of all microorganisms were found to be distributed log-normally. To improve the statistical models used in this study, the microorganism data employed as dependent variables were all log-transformed. One of the categories served as a reference group for comparison with the other groups. Each descriptive factor was given a value of zero when used as a reference variable.

## 3. Results

Temperature, relative humidity and carbon dioxide level (CO_2_) was shown by hospital in Table 2. The mean level of airborne bacteria (7.2 × 10^2^ CFU/m^3^) was lower than the indoor air quality standard (8.0 × 10^2^ CFU/m^3^) enforced by the Korean Environmental Protection Agency [11], while 30% of the 76 samples exceeded the standard. The average concentration of GNB was 1.7 × 10 CFU/m^3^, although 12 samples were below the limit of detection. The airborne fungi level ranged from 1.1 to 2.2 × 10^2^ CFU/m^3^. Significant differences were found among the six hospitals (*p* < 0.0001; Table 3). 

**Table 2:** Temperature, relative humidity and carbon dioxide level (CO_2_) by hospital.

Hospital	No. of Samples *	Temperature, °C		Relative Humidity, %		CO_2_, ppm	
		Mean ± SD	Range (min-max)	Mean ± SD	Range (min-max)	Mean ± SD	Range (min-max)
A	13	25.1 ± 0.4	24.5–25.9	66.8 ± 3.9	62.2–72.6	801 ± 221	458–1,061
B	12	26.5 ± 0.1	26.3–26.6	70.6 ± 1.7	67.6–72.8	432 ± 44	377–509
C	12	25.9 ± 0.4	25.1–66.3	65.7 ± 0.1	65.6–65.8	503 ± 87	393–625
D	12	25.8 ± 0.9	24.0–26.9	65.3 ± 0.7	63.5–65.8	481 ± 109	342–651
E	13	22.9 ± 0.8	21.7–24.1	65.8 ± 0.1	65.6–66.0	558 ± 111	366–692
F	14	20.7 ± 1.2	17.4–21.8	65.0 ± 0.4	64.4–65.6	409 ± 50	345–486
Total	76	24.4 ± 2.2	17.4–26.9	66.5 ± 2.5	62.2–72.8	531 ± 176	342–1,061

No of sample: number of time-weighted average (TWA) of data logged every 30 s during the sampling period of microorganisms.

**Table 3:** Airborne microorganism levels by hospital.

Hospital	No of sample	Bacteria, CFU/m^3^			GNB, CFU/m^3^			Fungi, CFU/m^3^		
		GM	GSD	Range (min-max)	GM	GSD	Range (min-max)	GM	GSD	Range (min-max)
A	13	8.7 × 10^2^	1.6	3.8 × 10^2^ – 1.8 × 10^3^	10	2.7	4 – 1.1 × 10^2^	53	1.9	21 – 2.1 × 10^2^
B	12	6.2 × 10^2^	3.2	50 – 2.3 × 10^3^	21	2.1	5 – 5.6 × 10	79	1.8	29 – 2.0 × 10^2^
C	12	7.6 × 10^2^	1.8	2.8 × 10^2^ – 1.8 × 10^3^	18	1.9	7 – 5.2 × 10	66	2.1	18 – 1.5 × 10^2^
D	12	6.8 × 10^2^	1.7	3.3 × 10^2^ – 1.8 × 10^3^	11	2.3	4 – 4.7 × 10	83	2.1	18 – 2.2 × 10^2^
E	13	5.4 × 10^2^	1.6	2.7 × 10^2^ – 1.7 × 10^3^	16	2.1	LOD – 4.0 × 10	59	1.9	25 – 2.1 × 10^2^
F	14	2.0 × 10^2^	1.7	80 – 4.5 × 10^2^	5	1.5	LOD – 1.1 × 10	36	2.7	11 – 1.5 × 10^2^
Total	76	5.5 × 10^2^	2.3	5.0 × 10 – 2.3 × 10^3^	13	2.4	LOD – 1.1 × 10^2^	59	2.2	11 – 2.2 × 10^2^

GM = geometric mean; GSD = geometric standard deviation, unitless; GNB = Gram-negative bacteria; LOD = Limit of detection (30 CFU/sample).

**Figure 1:**
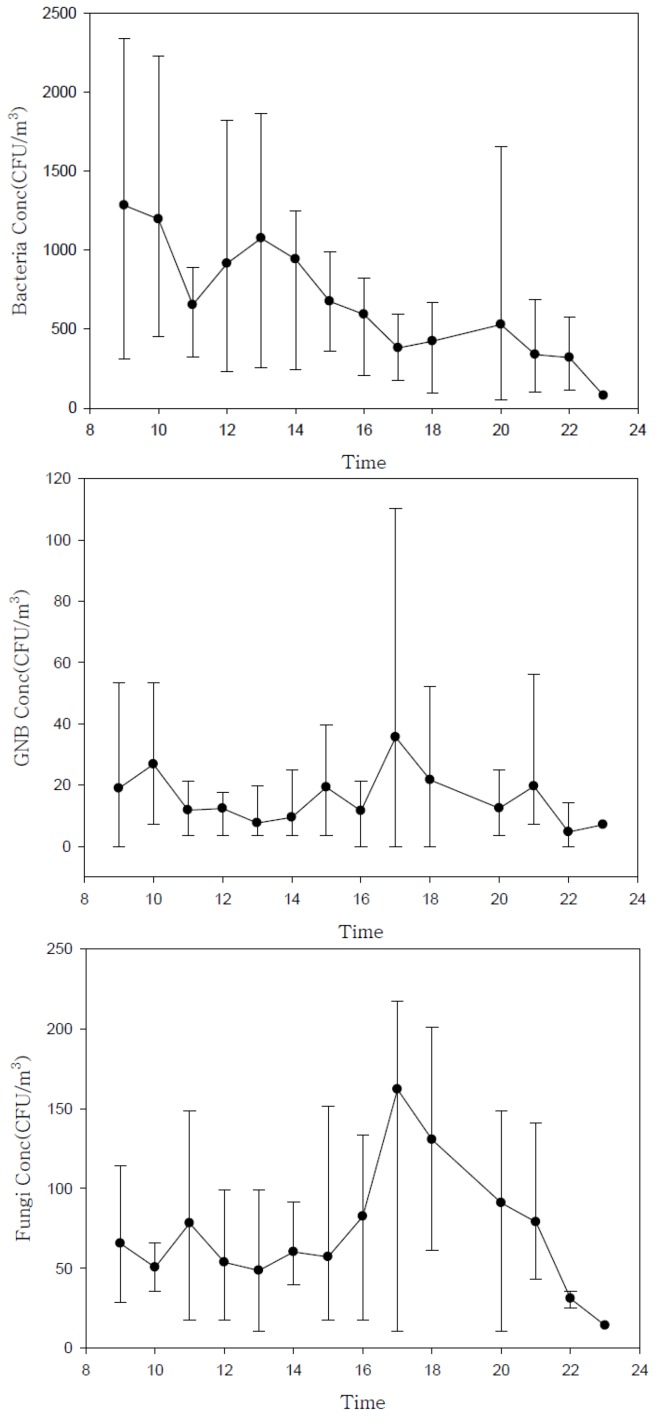
Daily variation in airborne microorganism concentrations (mean and range measured at six hospitals during summer, fall, and winter).

Our results indicate that the lobby air in all six hospitals was generally contaminated to some extent. We observed a clear pattern in microorganism levels over time, from the start of lobby service (08:00) to the end of lobby service, and after hours until midnight (Figure 1). 

**Figure 2:**
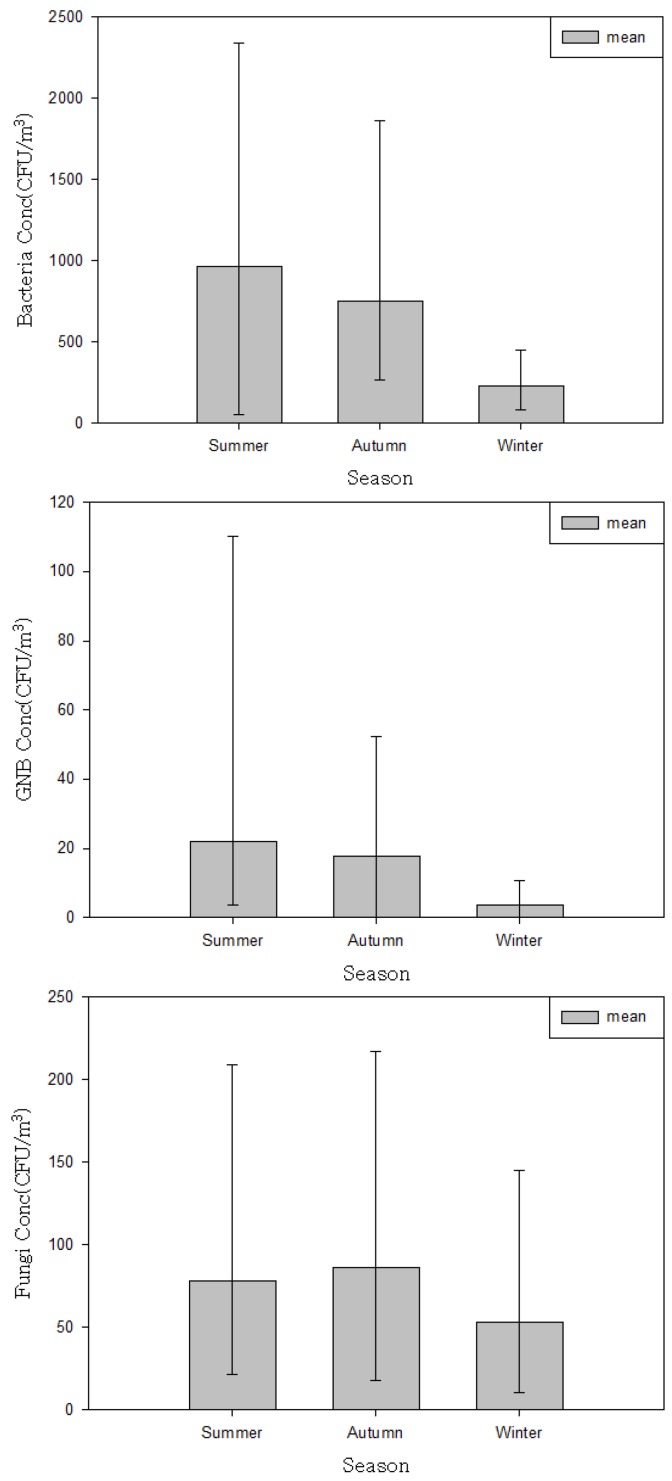
Variation in airborne microorganism concentrations by season.

The airborne bacteria level was higher during lobby service hours (between 08:00 and 18:00), when crowds of people were present and during summer (Figure 2). 

**Table 4:** Relationship between the levels of log-transformed airborne microorganism and quantitative variables.

Airborne microorganism level, CFU/m^3^	Relative humidity, %				Temperature, °C				Number of people present				CO_2_, ppm			
	β	SE	Model p	R^2^	β	SE	Model p	R^2^	β	SE	Model p	R^2^	β	SE	Model p	R^2^
Bacteria	NS	NS	NS	0.01	0.18	0.038	0.0001	0.23	0.01	0.001	<0.0001	0.37	0.01	0.001	<0.0001	0.37
GNB	0.05	0.04	0.2197	0.02	0.14	0.05	0.0068	0.1	NS	NS	NS	NS	NS	NS	NS	NS
Fungi	0.09	0.034	0.0127	0.08	NS	NS	NS	NS	0.002	0.001	0.056	0.05	0.002	0.001	0.056	0.05

GNB: Gram-negative bacteria; NS = not statistically significant at *p* = 0.25.

**Table 5:** The levels of log-transformed bacteria, gram-negative bacteria, and fungi among the categories of occupational and environmental variables.

Independent variables	No of sample	Bacteria, CFU/m^3^			GNB, CFU/m^3^			Fungi, CFU/m^3^		
		Mean	SD	ANOVA model p	Mean	SD	ANOVA model p	Mean	SD	ANOVA model p
Sampling time										
Service hours (08:00–18:00)	44	9.3 × 10^2^	5.7 × 10^2^	P ≤ 0.0001	1.5 × 10	1.3 × 10	P = 0.0422	5.6 × 10	3.3 × 10	p < 0.0001
After service hours (18:00–24:00)	32	4.4 × 10^2^	3.0 × 10^2^		2.0 × 10	2.2 × 10		1.1 × 10^2^	6.4 × 10	
Season										
Summer	25	9.7 × 10^2^	6.2 × 10^2^	p = 0.0001	2.2 × 10	2.4 × 10	p = 0.0005	7.8 × 10	5.4 × 10	p = 0.0206
Fall	37	7.5 × 10^2^	4.3 × 10^2^		1.8 × 10	1.3 × 10		8.6 × 10	5.6 × 10	
Winter	14	2.3 × 10^2^	1.1 × 10^2^		0.4 × 10	0.3 × 10		5.3 × 10	4.7 × 10	
Scale of hospital (number of beds)										
<1,000	52	6.3 × 10^2^	4.4 × 10^2^	p = 0.0192	1.3 × 10	1.7 × 10	p = 0.0016	7.3 × 10	5.6 × 10	p = 0.1365
≥1,000	24	9.3 × 10^2^	6.4 × 10^2^		2.4 × 10	1.6 × 10		8.7 × 10	5.1 × 10	
Lobby age (years)										
<10	24	9.3 × 10^2^	6.4 × 10^2^	p = 0.0001	2.4 × 10	1.6 × 10	p = 0.0059	8.7 × 10	5.1 × 10	p = 0.0788
10–19	27	4.1 × 10^2^	3.2 × 10^2^		1.0 × 10	1.2 × 10		6.3 × 10	5.1 × 10	
≥20	25	8.7 × 10^2^	4.4 × 10^2^		1.7 × 10	2.2 × 10		8.4 × 10	6.0 × 10	
Cleaning time										
Service hours (08:00–18:00)	37	8.7 × 10^2^	4.4 × 10^2^	p = 0.0001	1.8 × 10	1.9 × 10	p = 0.4623	8.3 × 10	5.7 × 10	p = 0.2351
After service hours (18:00–24:00)	26	5.7 × 10^2^	6.6 × 10^2^		1.4 × 10	1.7 × 10		7.1 × 10	5.3 × 10	
Location of fresh air inlet										
underground & 1st floor	52	6.8 × 10^2^	5.6 × 10^2^	p = 0.0446	1.6 × 10	1.9 × 10	p = 0.3524	7.0 × 10	5.2 × 10	p = 0.0902
higher than 2nd floor	24	8.3 × 10^2^	4.5 × 10^2^		1.8 × 10	1.3 × 10		9.3 × 10	5.7 × 10	
Number of filters in HVAC										
medium	14	2.3 × 10^2^	1.1 × 10^2^	p ≤ 0.0001	0.4 × 10	0.3 × 10	p = 0.0001	5.3 × 10	4.7 × 10	p = 0.0056
pre and medium	62	8.4 × 10^2^	5.2 × 10^2^		2.0 × 10	1.8 × 10		8.3 × 10	5.5 × 10	
Lobby volume (m^3^)										
<10,000	52	6.3 × 10^2^	4.4 × 10^2^	p = 0.0957	1.3 × 10	1.7 × 10	p = 0.0016	7.3 × 10	5.6 × 10	p = 0.1365
≥10,000	24	9.3 × 10^2^	6.4 × 10^2^		2.4 × 10	1.6 × 10		8.7 × 10	5.1 × 10	
Total	76	7.2 × 10^2 ^	5.3 × 10^2^		1.7 × 10	1.7 × 10		7.7 × 10	5.4 × 10	

GNB: Gram-negative bacteria

On the other hand, the reverse pattern was detected for fungi. Higher levels of fungi were detected after lobby service hours, when the HVAC system was not operating. Airborne microorganism levels were regressed against quantitative factors such as air temperature, humidity, CO_2_ levels, and the number of people in the lobby during sampling (Table 4), and were compared between qualitative hospital characteristic categories (Table 5). Simple linear regression indicated that air temperature, CO_2_ level, and the number of people in the lobby during sampling accounted for 23%, 25% and 37%, respectively, of the variation observed in the airborne bacteria level (*p* < 0.0001). The airborne GNB level was significantly associated only with air temperature (*p* = 0.068). Air temperature, humidity, and the number of people were found to affect the level of fungi at p < 0.25 (Table 4).

Regarding GNB, all qualitative variables except cleaning time (*p* = 0.4623) and location of fresh air supply (*p* = 0.3524) were significant at *p* < 0.05. The season and the number of filters in the HVAC were significantly associated with all three types of airborne microorganism (*p* ≤ 0.0001), being markedly higher in summer than in winter. Correlation matrix analysis revealed that airborne levels of GNB and fungi were significantly correlated with one another (Table 6).

**Table 6:** Correlation matrix analysis results between the log-transformed levels of airborne bacteria, gram-negative bacteria, and fungi levels (p-value)

	Bacteria	GNB	Fungi
Bacteria	1		
GNB	NS	1	
Fungi	NS	0.43 (p = 0.0002)	1

GNB: Gram-negative bacteria; NS = not statistically significant at *p* = 0.05.

All hospital and environmental variables identified as significant at *p* < 0.025 in the univariate analysis were included in the multiple regression analysis (Table 7). Season was the only factor that appeared to significantly affect airborne levels of all microorganisms. The other factors varied according to the type of microorganism. Significant statistical models were developed to predict airborne levels of bacteria, GNB and fungi accounting for 75% (*p* < 0.0001), 24% (*p* < 0.0001) and 36% (*p* < 0.0001) of the observed variation, respectively. 

## 4. Discussion

Most hospital-based airborne microorganism studies have been performed in clean rooms or departments, where the risk of infection is greatest [5,6,7]. Relatively little is known about microbial contamination in hospital lobbies. In the large hospitals included in our study, we found that the lobby air was contaminated with microorganisms, including bacteria, GNB, and fungi. The levels recorded (bacteria: 7.2 × 10^2^ CFU/m^3^; fungi: 5.5 × 10^2^ CFU/m^3^) were far higher than those hitherto reported in hospital clean rooms, such as operating rooms, hospital rooms, intensive care units (ICD), surgical units, hematological wards, pneumonological department and maternity wards [2,5,6,7]. Levels reported in hospital rooms with a high degree of cleanliness, such as operating rooms and transplant units, range from 0.1 to 10 CFU/m^3^. Augustowska and Dutkiewicz reported that mean monthly microflora measured in a hospital ward of the pneumonological department in Poland ranged from 257–436 CFU/m3 for airborne bacteria and from 10–96 CFU/m^3^, respectively [2].

**Table 7:** Multiple regression models for predicting the log-transformed levels of bacteria and GNB in a hospital lobby

Independent variable	Bacteria, CFU/m^3^			GNB, CFU/m^3^			Fungi, CFU/m^3^		
	Intercept	SE	p	Intercept	SE	p	Intercept	SE	p
Constant	2.79	2.13	0.195	2.43	0.18	0	NS		
Relative humidity, %	NS						0.10	0.04	0.035
Temperature, ℃	NS			NS			0.39	0.11	0.001
CO_2_, ppm	NS			NS			NS		
Number of people used	0.002	0.001	0.037	NS			NS		
Season									
Summer (reference)									
Fall	0.16	0.17	0.353	0.11	0.20	0.587	0.89	0.25	0.001
Winter	0.85	0.24	0.001	0.85	0.30	0.006	2.55	0.72	0.001
Sampling time									
Service hours (08:00–18:00) (reference)									
After service hours (18:00–24:00)	0.81	0.18	<0.0001	NS			NS		
Number of beds									
<1,000 (reference)									
>1,000	NS			0.49	0.20	0.019	NS		
Cleaning time									
Service hours (08:00–18:00) (reference)									
After service hours (18:00–24:00)	0.49	0.21	0.022	NS			0.61	0.34	0.079
Adjusted full model (R^2^)	0.75			0.24			0.36		
	(p ≤ 0.0001)			(p ≤ 0.0001)			(p ≤ 0.0001)		

GNB: Gram-negative bacteria; NS = not statistically significant at *p* = 0.05.

Our airborne bacteria level (7.2 × 10^2^ CFU/m^3^) was found to be higher than those (3.7 × 10^2^ CFU/m^3^ in hospital main lobby, 2.9 × 10^2^CFU/m^3^ in surgical ward and 2.0 × 10^2^CFU/m^3^ in ICD) measured in similar hospital in Korea using same monitoring method by Kim *et al*. [12]. 

Currently, there are no specific criteria in place to protect hospital lobby visitors from exposure to bio-aerosols. Such criteria should be developed to prevent potential adverse health effects, including respiratory and infectious diseases, in hospital users. In particular, although it may be difficult to establish that exposure to fungal aerosols occurs or that exposure presents a hazard, indoor fungal growth is inappropriate and should be removed [8]. Exposures to many bio-aerosols including microorganisms are virtually inevitable in indoor environments, including hospitals. Such exposure to higher levels than outdoors that we measured in hospital lobbies can be considered unavoidable, tolerable or acceptable for the majority of healthy persons. However, this is not to say that exposure of vulnerable lobby visitors or patients could be acceptable. The results of this study could be used to learn the lesson that such levels in hospital lobbies could be inappropriate and suitable action should be taken in order to protect the susceptible people who generally use hospital lobbies. Although the airborne microorganism levels reported here should not be directly interpreted as inhalation exposure because they were obtained from area samplers operating for only a short period, our results can be useful to assess airborne microorganism levels generated under various hospital lobby circumstances. With a better understanding of the factors that affect the levels of airborne microorganisms in hospital lobbies, effective control strategies can be established to reduce exposure to lobby users. In the present study, our selected hospital characteristics and environmental factors (Table 4, Table 5) were analyzed, and variables that significantly influenced the levels of airborne microorganisms were identified. Our findings may be useful as a guide for controlling or managing microorganism levels in hospital lobbies. To minimise airborne microorganism level, it is crucial to characterise the environmental determinants affecting the level of microorganisms generated in hospital lobby. First, special attention should be paid to hospital lobby air during the summer, and the highest levels of microorganisms should be carefully noted to minimize the effects of contamination. Season was the only factor found to be significantly associated with the airborne levels of all microorganisms. The levels of bacteria (9.7 × 10^2^ ± 6.2 × 10^2^ CFU/m^3^), GNB (2.2 × 10 ± 2.4 × 10 CFU/m^3^), and fungi (7.8 × 10 ± 5.4 × 10 CFU/m^3^) were significantly higher in summer than in either fall or winter (Table 5, Figure 2). This is likely due to the optimal weather conditions and high humidity. In particular, the mean bacteria levels measured during lobby service hours (08:00–18:00) in the summer and fall were 1.4 × 10^3^ CFU/m^3 ^and 9.1 × 10^2^ CFU/m^3^, respectively, both of which exceeded the indoor air quality standard for bacteria in Korea (8.0 × 10^2^ CFU/m^3^) (Figure 1). Bacterial contamination of lobby air was found to be high during times of peak lobby usage, compared to the measurements recorded after lobby service hours (18:00–24:00). This result is consistent with the regression analysis, which indicated that the number of people in the lobby during microbial sampling was significantly related to the airborne bacteria level (Table 7). The average number of people we monitored in the lobby during service time was 117 (range = 38–347). Reverse trends were detected for airborne fungi. Airborne fungi levels measured after service hours (when the HVAC system was turned off) during summer (1.1 × 10^2^ CFU/m^3^) and fall (1.2 × 10^2^ CFU/m^3^) were far higher than the measurements (5.5 × 10 and 5.6 × 10 CFU/m^3^) taken during lobby service hours. These results demonstrate that the presence of people may introduce bacteria (but not fungi) to hospital lobby areas, which is consistent with the fact that the main source of bacteria in indoor environments is people [4,13]. Temperature and humidity significantly affected fungi levels. During summer, when the humidity is high and there are many rainy days, the HVAC system (especially the filters) should be monitored and cleaned as frequently as possible. In all of the hospital lobbies examined, the HVAC system only recirculated lobby air (without drawing in outdoor air) during summer and winter. Building insulation, combined with poor ventilation, may also create an environment with elevated levels of bio-aerosols [1]. An HVAC system can act as a breeding ground for microorganisms, and then distribute biologically contaminated air throughout a building. All of the components in an HVAC system must be inspected, cleaned, drained, and/or replaced periodically. 

Second, if the input location of the ventilation system is found to contribute to the entry of outdoor air contaminants, it should be relocated. In four of the six hospitals we studied, the air intakes for the lobby HVAC were installed near the ground or underground. It is very common for a hospital to allow vehicles to pass directly in front of the hospital lobby. Having the air intake at or below ground level could facilitate the entry of outdoor contaminants, including vehicle exhaust, microorganisms, and excess moisture. Fresh air was not supplied through the HVAC system during summer and winter. However, because the lobby has several doors opening to the outside environment, outdoor air could be drawn into the lobby as people enter and leave. In the main entrance, which is the passageway between the hospital and its environment, the large numbers of patients, visitors and personnel raise the microbial rates especially at the afternoon because of the maximum activity of people there. The exchange between indoor and outdoor air raise the microbial rate brought from outside the hospital into the main entrance [16].

Third, special measures should be taken to reduce air contamination by GNB and fungi after 18:00, when lobby service ends. The highest level (mean 27.5 CFU/m^3^) of GNB was recorded after lobby service hours during summer. We found that five of the six hospitals turned off the HVAC system after lobby service hours. Increased GNB is associated with contaminated humidifiers, lower ventilation rates, the presence of cats and dogs, storage of food waste, and increased amounts of settled dust. GNB contamination in hospital lobbies may be related to the efficiency of ventilation and the presence of a contaminated humidifier. Unfortunately, the usage characteristics of humidifiers were not investigated in the present study. In addition, small commercial facilities, such as cafeterias, convenience stores, and snack bars, are commonly included in hospital lobbies. These facilities may also contribute to microbial contamination of the lobby. All hospital lobbies we studied had a cafeteria, which varied in size and style. Unfortunately, we did not examine the effect of size and style of cafeteria on airborne microorganisms.

Fourth, the filters used for lobby HVAC systems should have efficiencies >95% to adequately remove hazardous agents, including airborne microorganisms. All of the hospitals investigated in this study had independent HVAC systems with two-step filtering (pre and medium), which removes aerosols (including microorganisms) from outdoor air supplied to the lobby. According to the specifications of the medium filters installed in these systems, their filtration efficiencies range from 60% to 95%. None of the hospital lobbies in this study had HVAC systems with HEPA filters. For a medium efficiency filter, bacteria can be expected to have 60–90% penetration efficiency. Typically, airborne infectious microorganisms are adsorbed at the filter surface and then removed from the air [17]. The patient care area (PCA) ventilation system (including the lobby) is designed so that a fan provides 90–95% filtration (ASHRAE 52–92 Dust Spot Method) to the same PCA on each floor [18]. The installation of HEPA filters in the HVAC systems used for hospital lobbies could provide a level of protection against the entry of microorganisms comparable to that of hospital clean rooms. A laminar air-flow system, equipped with an HEPA filter that removes airborne particles of 0.3 µm and above with 99.97% efficiency, is generally used for orthopedic and other implant surgery facilities [19]. It is well known that HEPA filtration and positive pressure systems are an effective means of reducing microbial contamination in air. Such systems show a higher efficiency in reducing fungal contamination (200-fold) compared to bacterial contamination (5-fold) [5]. 

Fifth, floor cleaning times should be scheduled after lobby service ends. We found that floor cleaning or maintenance activities during lobby service hours significantly contributed to increased airborne bacteria and fungi levels (Table 5). We found that lobby floors were frequently either vacuumed by vacuum cleaner or wiped by mop during service time. Floor cleaning may cause particles deposited on the floor including microbes to disperse into air, which could contribute increase the level of airborne microorganism. The cleaning techniques employed should be effective for removing microorganisms. In addition, vacuum cleaners with HEPA filters should be used for cleaning to prevent aerosolization or regeneration of microorganisms on the floor. The airborne microorganism levels reported here should not be interpreted as direct inhalation exposure, because they were obtained from area samplers on specific days. 

A major limitation of this study is that it is not possible to know how representative our findings, obtained from small number of hospitals (n = 6), are with regard to hospital characteristics that have various types of lobby environment. Other environmental factors that we did not examine in this study may influence the level of airborne microorganisms in hospital lobby. Nevertheless, these results could be useful for identifying effective measures for reducing microbial loads. Our results are useful not only for characterizing the level of airborne microorganisms in hospital lobbies, but also for identifying specific factors that may significantly influence airborne concentrations of these agents, and recommending mitigation procedures. Based on our results, a number of engineering, administrative, and regulatory measures could appropriately be taken to reduce exposure to microorganisms in hospital lobbies. Hospitals may benefit from increased surveillance by government regulatory agencies, which currently take no special note of the fact that lobby users may be exposed to high levels of hazardous agents, including microorganisms.

In conclusion, we found that hospital lobby air was generally contaminated with microorganisms including bacteria, GNB, and fungi. Our results not only confirm that hospital lobbies are contaminated at various times of the day, but also identify specific factors that may significantly influence the airborne concentrations of these agents, and thus provide a starting point for the recommendation of mitigation measures.

## References

[1] Douwes J., Thorne P., Pearce N., Heederic D. (2003). Bioaerosol health effects and exposure assessment: Progress and prospects. Ann. Occup. Hyg..

[2] Augustowska M., Dutkiewicz J. (2006). Variability of airborne microflora in a hospital ward within a period of one year. Ann. Agric. Environ. Med..

[3] Scaltriti S., Cencetti S., Rovesti S., Marchesi I., Bargellini A., Borella P. (2007). Risk factors for particulate and microbial contamination of air in operating theatres. J. Hosp. Infect..

[4] Obbard J., Fang L. (2003). Airborne concentrations of bacteria in a hospital environment in Singapore. Water Air Soil Pollut..

[5] Ortiz G., Yagüe G., Segovia M., Catalán V. (2009). A study of air microbe levels in different areas of a hospital. Curr. Microbiol..

[6] Augustowska M., Dutkiewicz J. (2006). Variability of airborne microflora in a hospital ward within a period of one year. Ann. Agric. Environ. Med..

[7] Li C.S., Hou P.A. (2003). Bioaerosol characteristics in hospital clean rooms. Sci. Total Environ..

[8] Macher J. (1999). Bioaerosols: Assessment and Control.

[9] Andersen G. (1984). Single Stage/N6 Microbial Sampler.

[10] Park D.U., Ryu S.H., Kim S.B., Yoon C.S. (2011). An assessment of dust, endotoxin, and microorganism exposure during waste collection and sorting. J. Air Waste Manage. Assoc..

[11] Korean Ministry of Environment (2004). Air Quality for Public Building.

[12] Kim K.Y., Kim Y.S., Kim D. (2010). Distribution characteristics of airborne bacteria and fungi in the general hospitals of Korea. Ind. Health.

[13] Chow T.T., Yang X.Y. (2005). Ventilation performance in the operating theatre against airborne infection: Numerical study on an ultra-clean system. J. Hosp. Infect..

[14] Medrela-Kuder E. (2003). Seasonal variations in the occurrence of culturable airborne fungi in outdoor and indoor air in Cracow. Int. Biodeter. Biodegrad..

[15] Oliveira M., Ribeiro H., Abreu I. (2005). Annual variation of fungal spores in atmosphere of Porto: 2003. Ann. Agric. Environ. Med..

[16] Qudiesat K., Abu-Elteen K., Elkarmi A., Hamad M., Abussaud M. (2009). Assessment of airborne pathogens in healthcare settings. Agtivsn J. Microbiol. Res..

[17] Bomo A.M., Stevik T.K., Hovi I., Hanssen J.F. (2004). Bacterial removal and protozoan grazing in biological sand filters. J. Environ. Quality.

[18] Falvey D.G., Streifel A.J. (2007). Ten-year air sample analysis of *aspergillus* prevalence in a university hospital. J. Hosp. Infect..

[19] Dharan S., Pittet D. (2002). Environmental controls in operating theatres. J. Hosp. Infect..

